# Obesity Paradox in Cancer: A Complex Interplay Between Risk and Therapeutic Outcomes

**DOI:** 10.1002/mco2.70494

**Published:** 2025-11-18

**Authors:** Tong Chen, Wenjia Zhai, Jiaao Song, Wenqiang Liu, Zhenjie Wu, Fu Yang, Linhui Wang

**Affiliations:** ^1^ Department of Urology Shanghai Changhai Hospital, Naval Medical University Shanghai China; ^2^ Department of Medical Genetics Naval Medical University Shanghai China; ^3^ Shanghai Key Laboratory of Medical Bioprotection Shanghai China; ^4^ Key Laboratory of Biological Defense Ministry of Education Shanghai China

**Keywords:** body mass index, immune checkpoint inhibitors, metabolic dysregulation, obesity paradox, tumor microenvironment

## Abstract

Obesity is a recognized risk factor for cancer development and progression. Paradoxically, growing clinical evidence across several cancer types indicates that elevated body mass index (BMI) or specific body composition characteristics—such as increased visceral fat or preserved skeletal muscle—may be associated with moderately improved overall survival in patients undergoing immune checkpoint inhibitor (ICI) therapy. This seemingly contradictory phenomenon is often described as the “obesity paradox.” In this review, we delineate the etiological versus therapeutic implications of obesity, synthesizing data from non‐small cell lung cancer, renal cell carcinoma, melanoma, and hepatocellular carcinoma. Proposed explanations include low‐grade inflammation with signal transducer and activator of transcription 3‐mediated programmed death‐1 upregulation, insulin/insulin‐like growth factor‐1 signaling, adipokine imbalance, stromal fibrosis and hypoxia, and metabolic reprogramming that may alter T‐cell function and tumor immunogenicity. Nonbiological factors—including dosing strategies, sarcopenia, and sex‐specific differences—are also examined. We advocate for future research employing comprehensive body composition assessments, standardized pharmacokinetic/pharmacodynamic analyses, and consideration of sex and metabolic health. Clarifying the temporal and mechanistic basis of the obesity–ICI benefit relationship will inform the optimization of cancer immunotherapy.

## Graphical abstract

This review summarizes how obesity‐driven metabolic and immune alterations contribute to both cancer development and the paradoxical improvement of immunotherapy outcomes.

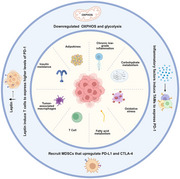

## 1 Introduction

Obesity is an increasingly severe public health issue worldwide and has been widely confirmed as a high‐risk factor for various chronic diseases. It is typically assessed by body mass index (BMI), though diagnostic thresholds vary geographically: the World Health Organization defines obesity as a BMI ≥ 30 kg/m^2^ [1]; however, different criteria are used in Asia. For example, in India, obesity is defined as a BMI ≥ 25 kg/m^2^, whereas in China, the threshold is a BMI ≥ 28 kg/m^2^ [2]. Obesity is closely associated with various diseases, including diabetes, cardiovascular disorders, arthritis, and cancers. These include breast cancer, renal cell carcinoma (RCC), and colorectal cancer (CRC) [3, 4, 5, 6, 7, 8]. Globally, approximately 3.6% of cancers (481,000 new cases annually) are related to elevated BMI [9]. Mechanistically, obesity drives carcinogenesis through systemic and tumor microenvironment (TME) inflammation, immune dysregulation, endocrine disturbances, and metabolic reprogramming [10], highlighting its detrimental impact on cancer etiology.

**FIGURE 1:**
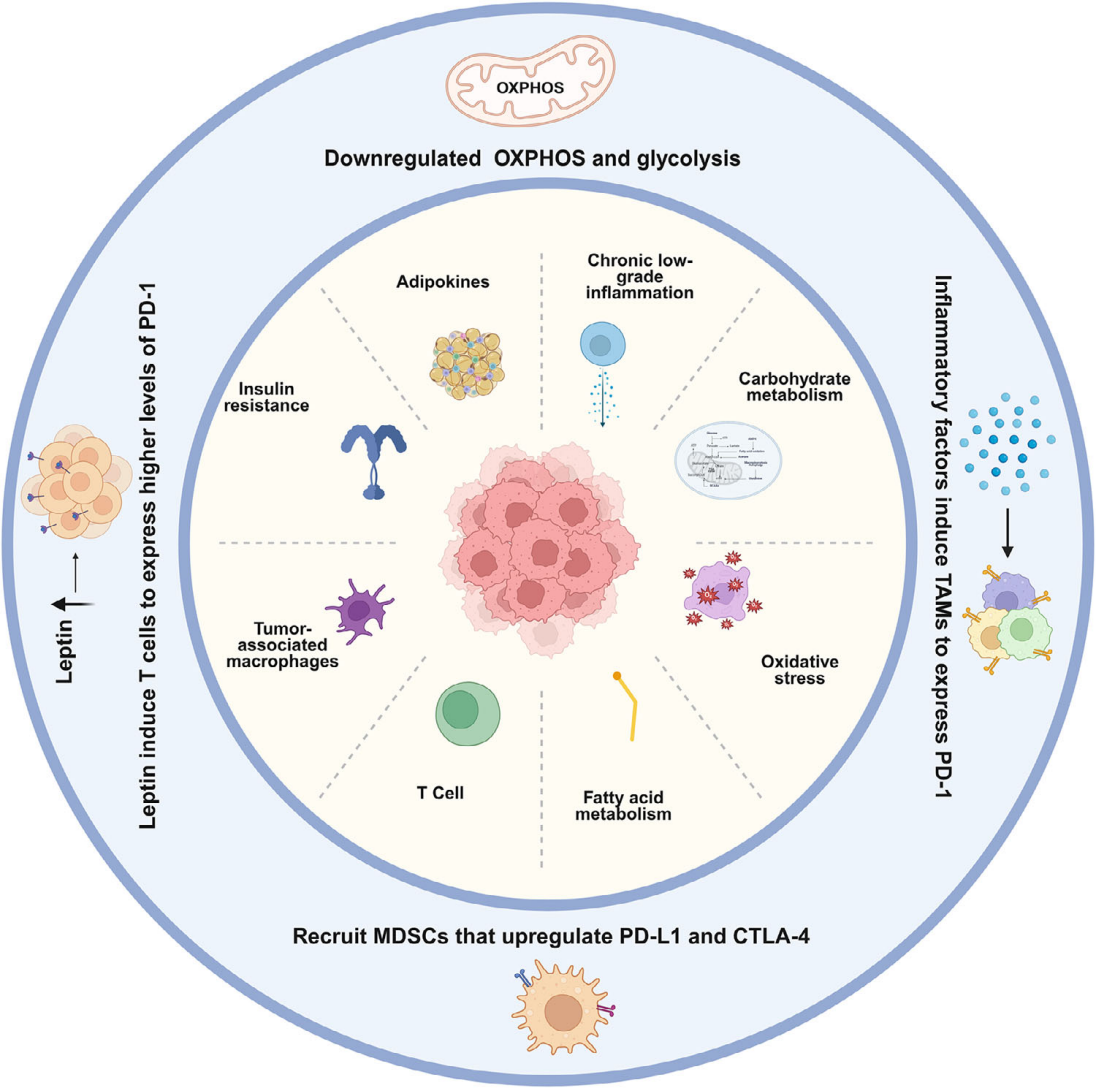
The impact of obesity on the tumor microenvironment and immune responses. Obesity induces changes in the tumor microenvironment through multiple mechanisms, including systemic and local chronic low‐grade inflammation; altered carbohydrate, fatty acid, and oxidative metabolism; and endocrine dysfunction mediated by adipokines such as leptin. These alterations drive the polarization of tumor‐associated macrophages (TAMs), insulin resistance, and metabolic disturbances. Obesity‐related leptin promotes programmed cell death protein 1 (PD‐1) expression on T cells, while inflammatory factors induce PD‐1 expression on TAMs and enhances the expression of PD‐L1 and CTLA‐4 on myeloid‐derived suppressor cells (MDSCs). Obesity‐associated metabolic reprogramming results in the downregulation of oxidative phosphorylation (OXPHOS) and glycolysis, which contribute to increased immune cell infiltration in peritumoral adipose tissue. Together, these dynamic interactions highlight the dual role of obesity in promoting cancer development while paradoxically influencing the efficacy of immune checkpoint inhibitor (ICI) therapy. *Abbreviations*: OXPHOS, oxidative phosphorylation; PD‐1, programmed cell death protein 1; PD‐L1, programmed death‐ligand 1; CTLA‐4, cytotoxic T lymphocyte‐associated protein 4; TAMs, tumor‐associated macrophages; MDSCs, myeloid‐derived suppressor cells; ICIs, immune checkpoint inhibitors.

Paradoxically, accumulating evidence indicates that some obese patients experience improved clinical outcomes after immune checkpoint inhibitor (ICI) therapy, a phenomenon termed the “obesity paradox” [11, 12, 13]. This counterintuitive association has been observed in various tumor types, including RCC [14] and malignant melanoma [15] (Table 1). Proposed mechanisms include chronic low‐grade inflammation that recruits and activates effector immune cells, thereby enhancing antitumor immunity [16, 17], as well as obesity‐related metabolic disturbances—elevated serum insulin, insulin‐like growth factor‐1 (IGF‐1), and adipokines—that may increase tumor immunogenicity and facilitate immune‐mediated tumor clearance [18, 19] (Figure 1). Nevertheless, the biological basis of this paradox remains incompletely understood.

**TABLE 1:** Impact of BMI and body composition on cancer risk and ICI outcomes in selected cancers.

	Body composition and tumor risk			Body composition and ICI outcomes		
Tumor type	Study (author, reference)	Measure	Effect (95% CI)	Study (author, reference)	Measure	ICIs outcome (OS/HR with 95% CI)
Lung cancer	Hidayat et al. [31]	+10 cm WC/+0.1 WHR	WC: RR 1.10 (1.04–1.17); WHR: RR 1.05 (1.00–1.11)	Zhang et al. [38]	BMI ≥ 25 vs. 18.5–24.9	HR 0.82 (0.74–0.90)
	Yu et al. [32]	+5 kg/m^2^ BMI	RR 1.09 (1.03–1.15)	Ichihara et al. [40]	BMI ≥ 22 vs. <22	OS 15.4 vs. 13.5 mo (*p* = 0.021)
RCC	Renehan et al. [8]	+5 kg/m^2^ BMI	RR 1.24(1.20–1.28)	Sanchez et al. [14]	BMI ≥ 30 vs. 18.5–24.9	HR 0.54 (0.31–0.95)
	Liu et al. [50]	+1 kg/m^2^ BMI	RR 1.06(1.05–1.06)	Lalani et al. [55]	BMI ≥ 25 vs. <25	HR 0.75 (0.57–0.97)
Malignant melanoma	Renehan et al. [8]	+5 kg/m^2^ BMI (men only)	RR 1.17 (1.05–1.30)	Richtig et al. [15]	BMI ≥ 25 vs. <25	HR 1.81 (0.98–3.33)
	Mehta and Hasija [66]	BMI > 25 vs. <25	OR 1.36 (1.20–1.55)	Jan et al. [75]	BMI ≥ 25 vs. <25	OS 71.7 vs. 36.7 month
HCC	Xiao et al. [86]	BMI ≥ 30 vs. <30	RR 1.31 (1.13–1.52)	Wang et al. [99]	BMI ≥ 25 vs. <25	OS 15.3 vs. 11.9 month
	Jun et al. [87]	BMI ≥ 30/+5 kg/m^2^ BMI	RR 1.60 (1.55–1.66)	Yamamoto et al. [100]	BMI ≥ 25 vs. <25	Median OS 599 d vs. NA (*p* = 0.034)

*Abbreviations*: BMI, body mass index; CI, confidence interval; d, days; HCC, hepatocellular carcinoma; HR, hazard ratio; ICIs, immune checkpoint inhibitors; mo, months; N/A, not applicable; OR, odds ratio; OS, overall survival; RCC, renal cell carcinoma; RR, relative risk; WC, waist circumference; WHR, waist‐to‐hip ratio.

Not all studies corroborate these observations: several large cohorts and meta‐analyses show neutral effects of obesity on ICI efficacy [20], and benefits often attenuate after adjusting for confounders such as nutritional status, skeletal muscle mass, or obesity‐related immune remodeling [16, 17, 21, 22]. Furthermore, nonbiological factors—including pharmacokinetic bias from fixed‐dose ICI regimens that yield relatively higher mg/kg exposure in heavier patients—may partially mimic a survival advantage. Heterogeneity in genetics, lifestyle, and comorbidities further complicates interpretation [23, 24, 25, 26]. Collectively, these findings underscore that any survival advantage is context‐dependent rather than universal.

Therefore, this review focus on cancers treated with ICIs, integrates both supporting evidence and contradictory findings, and outlines potential mechanistic hypotheses that may underlie the obesity paradox, with the hope of providing new insights for optimizing personalized treatment plans and improving patient prognosis.

## 2 Cancer Types Linked to the Obesity Paradox

To better elucidate the clinical manifestations of the obesity paradox, this section summarizes epidemiological evidence and treatment outcomes with ICIs across various cancer types, including lung cancer, RCC, malignant melanoma, and hepatocellular carcinoma (HCC).

### 2.1 Lung Cancer

#### 2.1.1 Epidemiology

Lung cancer is the leading cause of cancer‐related deaths worldwide, with approximately 127,070 deaths annually due to lung cancer [27]. Although obesity has been extensively studied as a risk factor for several cancers, its role in lung cancer remains unclear, according to the International Agency for Research on Cancer [28]. Most studies indicate that smoking is the primary risk factor for lung cancer [27], whereas obesity is often overlooked. However, with the increasing number of nonsmoking lung cancer patients, scholars have begun to focus on metabolic factors, such as obesity, and their potential impact on lung cancer [29].

Different obesity definitions may influence estimates of lung cancer incidence. A case‒control study revealed a negative association between lung cancer risk and BMI. The study included 2625 newly diagnosed lung cancer patients and 3381 control patients. Compared with men with a normal BMI, the adjusted odds ratios (ORs) for lung cancer were 2.7 (95% confidence interval [CI]: 1.2–6.2) for those with a BMI < 18.5 kg/m^2^, 0.9 (95% CI: 0.7–1.1) for those with a BMI of 25–29.9 kg/m^2^, 0.8 (95% CI: 0.6–1.1) for those with a BMI of 30–32.4 kg/m^2^, and 0.8 (95% CI: 0.6–1.0) for those with a BMI ≥ 32.5 kg/m^2^ (*p* = 0.02) [30]. However, abdominal obesity (such as waist circumference [WC] and the waist‐to‐hip ratio [WHR]) is associated with an increased risk of lung cancer. For every 10 cm increase in WC, the risk of lung cancer increased by approximately 10% (relative risk [RR] 1.10; 95% CI: 1.04–1.17), and for every 0.1 unit increase in WHR, the risk increased by 5% (RR = 1.05; 95% CI: 1.00–1.11) [31]. These findings suggest that abdominal fat may be a better predictor of lung cancer risk and that measuring WC and WHR, rather than BMI, may more effectively predict the relationship between obesity and lung cancer [31].

In studies of different types of lung cancer, the impact of obesity was significantly different. BMI is negatively associated with all types of non‐small cell lung cancer (NSCLC), particularly adenocarcinoma, which BMI is significantly negatively correlated with risk (per 5 kg/m^2^ increase, hazard ratio [HR] = 0.86, 95% CI: 0.84–0.89). However, BMI is positively associated with the risk of small cell lung cancer, with an HR of 1.09 (95% CI: 1.03–1.15) per 5 kg/m^2^ increase [32]. These findings suggest that obesity may affect lung cancer subtypes through distinct biological mechanisms.

Smoking is the primary risk factor for lung cancer and typically leads to weight loss, changes in body composition, and fat distribution [33]. Therefore, smoking may influence the effect of obesity on lung cancer [8]. A study indicated that being overweight (BMI 25–29.99 kg/m^2^) and class I obesity (BMI 30–34.99 kg/m^2^) were associated with a lower risk of lung cancer than a BMI of 23–24.99 kg/m^2^, with HRs of 0.76–0.93, respectively. The associations were similar in both men and women but were stronger in smokers than in never‐smokers (*p* = 0.006). Among never‐smokers, former smokers, and current smokers, the HRs for each 5 kg/m^2^ increase in BMI were 0.95 (95% CI: 0.90–1.00), 0.92 (95% CI: 0.89–0.95), and 0.89 (95% CI: 0.86–0.91), respectively. For never‐smokers, former smokers, and current smokers, the HRs for each 10 cm increase in WC were 1.09 (95% CI: 1.00–1.18), 1.12 (95% CI: 1.07–1.17), and 1.11 (95% CI: 1.07–1.16), respectively. When considered together, participants with low/normal BMI but high/very high WC had a 40.0% greater risk of lung cancer than did those with high BMI but normal/moderate WC. These findings suggest that the inverse association between BMI and lung cancer is not entirely due to the influence of smoking and that central obesity may be a factor that warrants more attention [32].

On the basis of summary statistics from the Genetic Associations and Mechanisms in Oncology consortium, the associations between genetic risk scores and each trait were tested, revealing that adult BMI is positively correlated with the overall incidence of lung cancer (OR = 1.27, 95% CI: 1.09–1.49 per standard deviation increase) [34]. Additionally, Mendelian randomization analysis revealed that BMI has a causal effect on lung cancer risk, particularly showing a stronger association with squamous cell carcinoma and small cell lung cancer [35].

The association between obesity and the incidence of lung cancer is still not well defined. Analyzing from different perspectives via various criteria may yield different results, and the influence of confounding factors may also play a role. Larger cohort studies that assess obesity using multiple criteria are needed for further research.

#### 2.1.2 Treatment Outcomes and Performance

In recent years, immunotherapies, represented by programmed death‐1 (PD‐1) and programmed death‐ligand 1 (PD‐L1), have gradually become the foundational treatments for advanced lung cancer [36, 37], and significant clinical progress has been achieved in NSCLC [36]. Many studies have indicated that obese lung cancer patients may exhibit better treatment outcomes when they are receiving immunotherapy [38, 39]. Overall, obesity has been associated with modestly improved survival on ICIs in NSCLC, yet effect sizes vary and some analyses are null.

In a meta‐analysis of NSCLC patients receiving ICIs, no significant differences were found in progression‐free survival (PFS) (HR = 0.885; 95% CI: 0.777–1.009, *p* = 0.068) or overall survival (OS) (HR = 0.947; 95% CI: 0.789–1.137, *p* = 0.560) between the low BMI group (BMI < 18.5 kg/m^2^) and the high BMI group (BMI ≥ 25 kg/m^2^). However, compared with patients with a normal weight (BMI 18.5–24.9 kg/m^2^), overweight and obese patients presented prolonged PFS (HR = 0.862; 95% CI: 0.760–0.978, *p* = 0.021) and OS (HR = 0.818; 95% CI: 0.741–0.902, *p* < 0.0001) [38]. These findings suggest that obese patients may achieve better survival outcomes during immunotherapy. A retrospective study in Japan analyzed 513 NSCLC patients receiving monotherapy with PD‐1/PD‐L1 antibodies. The researchers used a BMI of 22 kg/m^2^ as the cutoff point between the high and low BMI groups. The study revealed that patients with a high BMI had significantly longer survival periods than did those with a low BMI (PFS: 3.7 vs. 2.8 months; OS: 15.4 vs. 13.5 months, both *p* < 0.05) [40]. An analysis combining data from four international multicenter clinical trials demonstrated that overweight and obese patients receiving atezolizumab treatment had significantly improved OS rates compared with patients with a normal BMI (HR = 0.81, 95% CI: 0.68–0.95; HR = 0.64, 95% CI: 0.51–0.81). The presence of tumor PD‐L1 further strengthened this association. The survival advantage associated with being overweight or obese was greater for PD‐L1‐positive tumors than for PD‐L1‐negative tumors (overweight: HR = 0.73; 95% CI: 0.58–0.91 vs. obese: HR = 0.48; 95% CI: 0.34–0.66) [41]. However, a retrospective study from Japan indicated that among NSCLC patients receiving ICIs, being overweight or obese was associated with a lower risk of death than being in lower BMI categories. This relationship exhibited a U‐shaped pattern, with the lowest risk observed in individuals with a BMI between 25 and 34 kg/m^2^ [42].

The survival advantage associated with obesity may extend beyond ICI therapy. Previous observations have indicated that a high BMI is associated with better outcomes in surgery, radiotherapy, and certain types of chemotherapy for both early and advanced NSCLC patients [43, 44, 45, 46]. Overall, although existing evidence suggests that obese patients may benefit more from immunotherapy, treatment plans still need to be tailored to individual circumstances, and potential risks during the treatment process should be closely monitored. Taken together, current evidence suggests that while obesity may confer modest survival benefits during ICI therapy, especially in NSCLC, prospective studies incorporating both BMI and central obesity metrics (WC, WHR) are required to clarify mechanisms and guide individualized treatment.

### 2.2 Renal Cell Carcinoma

#### 2.2.1 Epidemiology

RCC is a heterogeneous malignancy originating from renal tubular epithelium. The American Cancer Society estimates 81,800 new RCC cases and 14,890 deaths in the United States in 2023 [27]. RCC arises from renal tubular epithelium and comprises diverse histological subtypes, with clear cell RCC accounting for 90% of cases [47]. The occurrence of RCC is influenced by multiple factors, the most significant of which include age, sex, and race. For sporadic RCC, the known primary risk factors include smoking, long‐term use of analgesics [48], and renal diseases such as hypertension and chronic kidney disease [47]. The global obesity epidemic has emerged as a major RCC risk factor, particularly in populations with high BMI [49].

A meta‐analysis of 17 studies indicated that for every 5 kg/m^2^ increase in BMI, the risk of RCC incidence in males increased by 24% (RR = 1.24, 95% CI: 1.15–1.34), whereas in females, the risk increased by 34% (RR = 1.34, 95% CI: 1.25–1.43) [8]. Another large‐scale meta‐analysis encompassing 24 studies and over 8 million participants further confirmed the positive correlation between BMI and RCC risk. The study revealed a linear dose‒response relationship between BMI and RCC risk, with each 1 kg/m^2^ increase in BMI associated with a 6% increase in risk (RR = 1.06, 95% CI: 1.05–1.06). Additionally, for males, each 1 kg/m^2^ increase in BMI was associated with a 5% increase in RCC risk (RR = 1.05), whereas for females, the increase was 6% (RR = 1.06) [50].

In a retrospective study of 23 million East Asian individuals in Korea, researchers explored the relationships of general obesity (defined by BMI) and abdominal obesity (defined by WC) with the incidence of RCC. The study revealed that when a BMI ≥ 25 kg/m^2^ was used as the standard for obesity, general obesity was associated with a 32% increased risk of RCC diagnosis (HR = 1.32, 95% CI: 1.28–1.36). Further analysis revealed that for every 5 cm increase in WC, the risk of RCC incidence increased by 12.5% [51]. Additionally, in a multicenter retrospective study, researchers reported a significant correlation between an increase in visceral adipose tissue (VAT) and the occurrence of RCC. For every 10 cm^2^ increase in visceral fat area, the OR for RCC occurrence was 1.006 (6% increase in the odds of having clear cell RCC [ccRCC]) [52].

In summary, obesity and overweight are significantly associated with an increased risk of RCC. Multiple meta‐analyses and large‐scale studies have further confirmed the positive correlation between increased BMI and RCC risk, with this relationship being particularly significant in males. Furthermore, increases in WC and VAT have also been shown to be significantly associated with RCC incidence, suggesting that abdominal obesity and visceral fat may play important roles in the development of RCC.

#### 2.2.2 Treatment Outcomes and Performance

Beyond disease incidence, a growing body of evidence has examined how obesity shapes clinical outcomes in RCC, especially in the era of ICIs. Approximately one‐third of RCC patients present with distant metastasis at initial diagnosis, and among those with localized RCC, approximately 20–50% of patients still develop distant metastasis even after undergoing nephrectomy [53]. With the approval of the PD‐1 inhibitor nivolumab, treatment strategies for metastatic RCC have undergone significant changes. The use of ICIs in combination with or as monotherapy with targeted treatments has become the standard first‐line treatment regimen for metastatic ccRCC [53].

Overall, higher BMI often correlates with improved OS on ICIs in metastatic RCC, although effects on time to treatment failure (TTF) or objective response rate (ORR) and adjusted estimates are inconsistent across cohorts. A cohort study of pancancer data revealed that the relationship between obesity and ICI response varies among different cancer types. The largest difference in response rates was observed in RCC, where obese patients had an ICI response rate of 50.00% (39 out of 78) compared with 30.28% (43 out of 142) in nonobese patients (OR = 0.44; 95% CI: 0.24–0.80) (*p* = 0.01) [54]. This retrospective cohort study included 203 patients with metastatic clear cell RCC who received ICI therapy. The unadjusted analysis revealed that obese patients had significantly improved OS, with an HR of 0.54 (95% CI: 0.31–0.95), compared with patients with a normal BMI. However, after adjusting for RCC prognostic risk scores, this association disappeared, with an HR of 0.72 (95% CI: 0.40–1.30) [14]. In an analysis by the International Metastatic RCC Database Consortium, 735 patients with metastatic RCC receiving ICI therapy were included. The results indicated that patients with a BMI ≥ 25 kg/m^2^ had significantly improved OS after immunotherapy (HR = 0.75, 95% CI: 0.57–0.97). However, overweight and obese patients did not show significant improvements in TTF/ORR (TTF HR = 0.98, 95% CI: 0.80–1.20) [55]. The ARON‐1 project studied 675 patients with metastatic RCC receiving first‐line ICI combination therapy (or combination with targeted therapy). The results revealed that patients with a BMI > 25 kg/m^2^ derived greater overall clinical benefits than those with a BMI ≤ 25 kg/m^2^. Specifically, obese patients had a significantly longer OS, at 55.7 months, than they did at 28.4 months (*p* < 0.001). However, the impact of BMI on PFS was relatively weak. Although PFS increased (15.9 vs. 14.1 months), it did not reach statistical significance (*p* = 0.066) [56]. Additionally, a meta‐analysis of 2,281 metastatic RCC patients receiving ICI therapy revealed that patients with overweight and obese BMIs had significant improvements in both OS and PFS. Specifically, overweight and obese patients had significantly improved OS (HR = 0.77, 95% CI: 0.65–0.91) and PFS (HR = 0.66, 95% CI: 0.44–1.00) [57].

In addition to using BMI as a standard for measuring body composition, imaging methods have also been employed to assess body composition. A retrospective study evaluated the body composition imaging measurements of 205 metastatic ccRCC patients receiving ICI therapy. The study revealed that a higher skeletal muscle index (SMI) was significantly associated with improved OS (HR for low SMI vs. high SMI = 1.65, 95% CI: 1.13–2.43). However, there was no significant association between the obesity index and OS. Body composition variables were not associated with the ORR or PFS [58]. Another retrospective study involving 99 patients receiving first‐line nivolumab combined with ipilimumab therapy revealed that body composition parameters were not related to OS, but low SMI and subcutaneous obesity were associated with improved PFS [59]. Notably, the benefit attenuated after risk adjustment in some series, underscoring potential confounding.

Although these studies revealed correlations between obesity and treatment prognosis, several limitations remain. For example, BMI was not systematically evaluated as a continuous variable, and confounding factors such as smoking may have influenced the results. Many studies lacked longitudinal BMI measurements, which limited the assessment of BMI–treatment associations. Therefore, future research should consider these variables more precisely and conduct more precise analyses to validate these results.

### 2.3 Malignant Melanoma

#### 2.3.1 Epidemiology

Malignant melanoma is a type of tumor that originates from melanocytes, the pigment‐producing cells in the skin. Malignant melanoma is highly invasive and metastasizes rapidly [60]. The key prognostic factors for malignant melanoma primarily include Breslow thickness, the mitotic rate, ulceration status, patient age, sex, and the anatomical location of the tumor [60, 61, 62]. Epidemiological studies identify obesity as an important melanoma risk factor [63]. Epidemiological data show higher melanoma incidence and mortality in males than in females [60, 64]. These observations suggest that sex‐specific mechanisms influence melanoma development and treatment response. This may involve interactions between fat metabolism and sex hormones, particularly differential expression of androgen receptors (ARs) and estrogen receptors [65].

A meta‐analysis of 221 datasets examined the relationship between BMI and the incidence of malignant melanoma in males and revealed that for every 5 kg/m^2^ increase in BMI, the risk of malignant melanoma in males increased by 17% (RR = 1.17, *p* = 0.004) [8]. Another meta‐analysis of 2304 melanoma patients and 2468 controls revealed a positive association between a BMI greater than 25 kg/m^2^ and melanoma risk (OR = 1.36; 95% CI: 1.20–1.55). This study also indicated that a higher BMI is correlated with an increased incidence of melanoma [66]. In Sweden's SOS cohort (2007 obese patients, 2040 controls), bariatric surgery reduced melanoma risk by 57% [67].

In summary, epidemiological studies suggest a positive correlation between obesity and melanoma risk, especially in individuals with BMI > 25 kg/m^2^, although several studies report only weak associations [28, 68]. These discrepancies may be related to multiple confounding factors, including sex differences [69], menopausal status [70], and ultraviolet exposure [71], among others. Therefore, although obesity likely influences melanoma development, further research is needed to elucidate its mechanisms and clarify this relationship.

#### 2.3.2 Treatment Outcomes and Performance

Recent studies have shown that higher BMI may improve survival in melanoma patients receiving targeted therapy or immunotherapy [18, 20, 72, 73]. In an Austrian cohort of 76 metastatic patients treated with ipilimumab, a BMI ≥ 25 was associated with a higher objective response and longer OS (HR = 1.81, 95% CI: 0.98–3.33) [15]. Another retrospective study of metastatic melanoma evaluated BMI in relation to PFS and OS across multiple treatment types. The results revealed that among 538 metastatic malignant melanoma patients receiving ICIs, obesity was associated with a 25% reduction in disease progression risk and a 36% reduction in mortality risk. Sex‐stratified analysis showed stronger benefit in obese men (PFS HR = 0.62; OS HR = 0.62) than in women [74].

In 2078 ICI‐treated patients, OS was significantly longer in the 1412 with BMI ≥ 25 than in those with BMI < 25. Overweight patients had a median OS of 71.7 months, whereas nonoverweight patients had a median OS of 36.7 months (*p* < 0.001). This survival advantage persisted after propensity matching (median OS 67.7 vs. 36.7 months, *p* < 0.001) [75].

A retrospective study of 139 advanced cases assessed BMI and serum creatinine as a surrogate for muscle mass. The study revealed that in overweight or class I obese patients (BMI 30–34.9 kg/m^2^), there was a significant improvement in PFS (HR = 0.43, 95% CI: 0.19–0.95) and OS (HR = 0.26, 95% CI: 0.10–0.71). Multivariable modeling confirmed that overweight or class I obese patients with serum creatinine >0.9 had the longest survival. Increased skeletal muscle mass may partly explain this obesity paradox [76].

Another CT‐based body‐composition study of 84 patients treated with ipilimumab assessed clinical outcomes. The results indicated that baseline sarcopenia (reduced muscle mass) and low muscle quality were not significantly associated with shorter survival periods. However, as treatment progresses, a decrease in muscle mass might affect survival time [77]. Additionally, another retrospective study revealed that sarcopenic obesity was significantly associated with PFS (HR = 1.47, 95% CI: 1.02–2.12, *p* = 0.037) [78].

In summary, recent studies have demonstrated a significant association between BMI and survival outcomes in malignant melanoma patients undergoing immunotherapy. In particular, overweight and obese patients often exhibit longer OS and lower PFS when receiving ICIs. An increase in skeletal muscle mass may be a potential mechanism for the obesity paradox, explaining the survival advantage observed in obese patients during treatment. Sarcopenia strongly influences outcomes, underscoring the value of routine body‐composition assessment. Nevertheless, further research is needed to clarify the causal relationship between BMI and immunotherapy outcomes and to explore the specific impacts of different body compositions on treatment efficacy.

### 2.4 Hepatocellular Carcinoma

#### 2.4.1 Epidemiology

HCC is the most common primary liver cancer, arising from hepatocytes and accounting for 75–85% of cases worldwide. In 2022, there were 865,269 new cases of liver cancer globally, accounting for 4.3% of all new cancer cases. Chronic infection with the hepatitis B virus (HBV) and the hepatitis C virus (HCV), as well as long‐term alcohol consumption, are major risk factors for HCC. However, recent global data—especially from Western countries—show that metabolic dysfunction‐associated steatotic liver disease (MASLD) and steatohepatitis (MASH), often linked to metabolic syndrome or diabetes, are rapidly emerging as leading causes of HCC [79, 80, 81, 82, 83]. Multiple studies demonstrate a positive association between obesity and MASLD/MASH‐related HCC [84, 85].

A pooled analysis of 31 studies (>1.02 million participants) across North America and Asia, researchers reported that overweight (for Asians: 23 kg/m^2^ ≤ BMI < 30 kg/m^2^; for non‐Asians: 25 kg/m^2^ ≤ BMI < 30 kg/m^2^) or obese (BMI ≥ 30 kg/m^2^) MASLD patients had a significantly increased risk of developing HCC (RR = 1.31, 95% CI: 1.13–1.52, *I* ^2^ = 73%) [86]. Another prospective study followed 14.3 million Koreans for an average of 13.7 years and reported that, compared with individuals with a BMI ranging from 23.5–24.9 kg/m^2^, the risk of HCC significantly increased with increasing BMI. Specifically, for individuals with a BMI ≥ 25 kg/m^2^, a 5 kg/m^2^ increase in BMI was associated with an HR of 1.60 for the overall population, 1.60 for males, and 1.59 for females [87].

Beyond BMI, body fat percentage, WC, WHR, and VAT also show significant causal links to higher HCC risk [88, 89]. Obesity not only directly influences the occurrence of HCC but also synergizes with other risk factors, such as viral infections and alcohol consumption, to promote HCC development. For example, after data from two multicenter prospective cohort studies involving 1911 patients in the United States were analyzed, researchers reported that obese cirrhotic patients carrying the patatin‐like phospholipase domain‐containing protein 3 (PNPLA3) G allele variant presented significantly increased risks of HCC development. Specifically, statistical analysis revealed that obese carriers faced a 2.4‐fold greater risk of HCC than nonobese variant carriers did (HR = 2.40, 95% CI: 1.33–4.31). Notably, heavy alcohol consumption synergized with obesity to promote both initiation and progression of HCC, independent of PNPLA3 G allele status [90]. Another study analyzed 2733 liver cirrhosis patients (average age 60.1 years, 31.3% female) and reported that patients with MASLD‐related cirrhosis had a lower risk of progressing to HCC than did those with cirrhosis caused by HBV or HCV infection. However, overweight/obese patients had a significantly increased risk of HCC (HR = 1.79, 95% CI: 1.08–2.95) [91].

Metabolic abnormalities may be a prerequisite for obesity to promote the occurrence of HCC. For individuals with a low BMI (BMI < 25 kg/m^2^), the linear association between obesity and the occurrence of HCC is not statistically significant [87]. Approximately 40% of MASLD patients are not obese; however, these patients have a relatively high mortality rate [92]. The study revealed that, compared with metabolically healthy individuals (i.e., those without dyslipidemia, hypertension, or diabetes), metabolically unhealthy individuals who were overweight (OR = 1.89, 95% CI: 1.31–2.72) or obese (OR = 1.50, 95% CI: 1.07–2.09) had a significantly greater risk of HCC, with a significant interaction between metabolic health and obesity status (interaction *p* value = 0.0411) [88].

Additionally, some studies have noted that obesity, as an environmental and metabolic risk factor, has significantly different effects on males and females. Central obesity was associated with a greater risk of HCC in males (HR = 1.65, 95% CI: 1.18–2.31) but not in females. This sex‐specific association may be attributed to differences in fat distribution between males and females, with males typically exhibiting abdominal obesity and females tending to accumulate more fat in the hip and thigh regions, leading to varying susceptibilities to liver diseases [93]. In females, obesity during pregnancy may also alter the gut microbiota, increasing offspring susceptibility to HCC [94, 95].

In summary, obesity is a significant risk factor for the development of HCC, particularly in the context of MASLD and MASH. Obesity promotes the occurrence of HCC not only by directly altering the tumor immune microenvironment [89] but also by enhancing the pathogenic effects of other risk factors, such as alcohol consumption and viral infections. However, for metabolically normal overweight or obese individuals, the risk of developing HCC does not significantly increase.

#### 2.4.2 Treatment Outcomes and Performance

HCC management is stage‐ and liver‐function‐dependent and includes surgery, targeted therapy, and immunotherapy. For early‐stage liver cancer patients, surgical resection is the preferred curative treatment. In recent years, ICIs have transformed the treatment of advanced HCC; combinations such as atezolizumab plus bevacizumab or durvalumab plus tremelimumab are now the standard of care [96, 97].

Obesity may independently affect immunotherapy efficacy by upregulating immune‐related genes [98]. In a study of HCC patients with HBV infection, the efficacy of lenvatinib combined with carrelizumab was evaluated. The results revealed that overweight (BMI: 25–30 kg/m^2^) and obese (BMI ≥ 30 kg/m^2^) patients had significantly longer PFS and OS than did normal‐weight patients (PFS: 8.53 vs. 6.30 months, *p* < 0.001; OS: 15.30 vs. 11.90 months, *p* = 0.001) [99].

A retrospective analysis of the impact of atezolizumab combined with bevacizumab therapy in patients with unresectable HCC included 67 patients receiving immunotherapy. The study indicated that patients with a high BMI (BMI ≥ 25 kg/m^2^) had longer OS than did those with a low BMI (BMI < 25 kg/m^2^) did (median OS: not applicable [NA] days vs. 599 days, *p* = 0.034), although there was no significant difference in PFS (median PFS: 168 vs. 165 days, *p* = 0.571) [100].

Another study investigated the effect of BMI on combination therapy with atezolizumab and bevacizumab in patients with HCC. The results revealed that HCC patients with a BMI ≥ 25 had similar survival periods when receiving this treatment compared with patients with a normal or low BMI [101].

A study encompassing 305 HCC patients receiving immunotherapy revealed that the VAT index (VATI) had a significant protective effect on the OS of obese patients. For every 10 cm^2^/m^2^ increase in VATI, mortality rates in obese, normal‐weight, and underweight patients decreased by 17.7, 13.7, and 2.9%, respectively. High VATI, high SMI, and skeletal muscle density (SMD) were significantly associated with better OS, and these associations did not significantly differ across BMI subgroups or sexes. These findings suggest that high VATI and moderate BMI may have a protective effect on the survival of HCC patients receiving immunotherapy [102].

Another study, which used different evaluation criteria, assessed sarcopenia and myosteatosis through preimmunotherapy CT scans and included 138 patients receiving immunotherapy for advanced HCC. The results revealed that patients with sarcopenia had significantly poorer PFS (*p* = 0.048) and OS (*p* = 0.002). Additionally, sarcopenic obese patients (i.e., BMI > 25 kg/m^2^ with sarcopenia) had significantly worse OS than did overweight patients without sarcopenia (*p* = 0.006) [103]. This study supports the importance of muscle mass (especially sarcopenia) as a prognostic factor for immunotherapy efficacy, indicating that even obese patients may experience adverse immunotherapy outcomes if they have sarcopenia. Therefore, in clinical practice, BMI, muscle mass, and fat distribution should be comprehensively considered when evaluating the efficacy of immunotherapy and developing more personalized treatment plans.

In summary, current evidence on the relationship between obesity and ICI efficacy in HCC remains inconclusive, with conflicting findings across retrospective cohorts. Limitations include small sample sizes, incomplete data, and methodological biases. Well‐designed prospective trials are needed to clarify whether and how obesity influences ICI outcomes and to guide individualized therapy.

## 3 Biological Foundations of Obesity as a Cancer Risk Factor

Understanding the biological mechanisms through which obesity promotes cancer development is a critical component in interpreting the obesity paradox. Obesity triggers a series of systemic and cellular alterations—including metabolic, hormonal, and immune changes—that collectively contribute to a tumor‐promoting microenvironment. This section summarizes the key biological foundations of obesity‐driven carcinogenesis, with a focus on chronic inflammation, metabolic reprogramming, endocrine abnormalities, and immune dysregulation.

### 3.1 Pathophysiological Changes in Obesity Driving Cancer Development

#### 3.1.1 Immune Cell Infiltration and Chronic Inflammatory Factors

Obesity is widely studied as a state of chronic, low‐grade inflammation characterized by the excessive expansion and dysfunction of adipose tissue, which drives immune cell infiltration and the elevation of proinflammatory factors. In obese individuals, the expansion and apoptosis of adipocytes recruit various immune cells, including macrophages, T cells, and neutrophils, to infiltrate the adipose tissue [104]. During this process, macrophages transition from the anti‐inflammatory M2 phenotype to the proinflammatory M1 phenotype, releasing cytokines such as tumor necrosis factor‐alpha (TNF‐α), interleukin‐6 (IL‐6), and monocyte chemoattractant protein‐1. As a key proinflammatory cytokine, TNF‐α can either trigger apoptosis or promote cell survival, depending on the balance between caspase activation and the nuclear factor‐κB (NF‐κB) signaling pathway [105]. Mechanistically, TNFR1 signaling bifurcates into a membrane‐bound complex I that favors survival via NF‐κB/mitogen‐activated protein kinase (MAPK) and a cytosolic complex II (with Fas‐associated death domain and caspase‐8) that licenses death; ubiquitin scaffolds (cellular inhibitor of apoptosis proteins 1/2, linear ubiquitin chain assembly complex) and kinase checkpoints (IκB kinase alpha/beta and TANK‐binding kinase 1/IκB kinase epsilon phosphorylation of RIPK1) keep the balance toward survival, while NF‐κB‐driven FLIP and caspase‐8 cleavage of RIPK1 act as additional brakes. When these checkpoints are disabled, TNF signaling shifts to caspase‐8‐driven apoptosis or receptor‐interacting protein kinase 1/3 (RIPK1/RIPK3)/mixed lineage kinase domain‐like protein‐dependent necroptosis—and in some contexts, caspase‐8 can also cleave gasdermin D to trigger pyroptosis—releasing damage‐associated molecular patterns that amplify inflammation and barrier dysfunction [106]. These circuitries explain why pharmacologic targeting of RIPK1 or death‐pathway components is being explored as an adjunct or alternative in TNF‐driven disease. IL‐6 and its receptor promote antiapoptotic and proliferative effects through the Janus kinase 2 signaling pathway [107]. IL‐6 also activates the phosphoinositide 3‐kinase/protein kinase B (PI3K/AKT) pathway, promoting the proliferation and survival of cancer cells [108]. Moreover, interferon‐gamma (IFN‐γ) secreted by cluster of differentiation 8 positive T cells (CD8^+^ T cells) further amplifies inflammatory signaling [109, 110]. The expression of these inflammatory cytokines can further impact the immune microenvironment. For instance, TNF‐α and IFN‐γ can enhance the expression of major histocompatibility complex class II molecules, thereby augmenting antigen presentation [111]. Additionally, TNF‐α and IL‐6 can facilitate the homing of immune cells and promote antitumor immunity by upregulating the expression of adhesion molecules such as intercellular adhesion molecule‐1 and vascular cell adhesion molecule‐1, as well as chemokines like C‐X‐C motif chemokine ligand 10 (CXCL10) and C‐C motif chemokine ligand 2 (CCL2) [112, 113]. The latest single‐nucleus RNA sequencing and spatial transcriptomics analyses have also demonstrated that obesity can induce the recruitment of immune cells such as macrophages and lymphocytes, and shift the microenvironment to a proinflammatory state. Compared with the obese group, the fat‐associated macrophages in the weight loss group shift toward a less inflammatory subtype, and the levels of proinflammatory chemokines (C‐X‐C motif chemokine ligand 2 (CXCL2), CCL2, and IL‐6) and putative stress signals (transforming growth factor beta 1, amphiregulin, nicotinamide phosphoribosyltransferase, and thrombospondin‐1) in the microenvironment are significantly reduced, indicating that weight loss can reverse the inflammation and stress caused by obesity [114].

Chronic low‐grade inflammation is tightly linked to many cancers, influencing tumor initiation and progression through several mechanisms. First, proinflammatory factors activate tumor pathways such as signal transducer and activator of transcription 3 (STAT3) and NF‐κB, stimulating cell proliferation and inhibiting apoptosis, which enhances tumor cell survival and growth [115]. Second, chronic inflammation alters the tissue microenvironment by upregulating angiogenic factors, such as vascular endothelial growth factor, which promotes angiogenesis and increases the recruitment of immunosuppressive cells (e.g., regulatory T [Treg] cells, myeloid‐derived suppressor cells [MDSCs], and tumor‐associated macrophages [TAMs]), thereby supporting tumor survival and invasion [116]. Additionally, the oxidative stress and excessive reactive oxygen species (ROS) generated by chronic inflammation cause DNA damage, genetic mutations, and chromosomal instability, creating a genetic foundation for cancer development [117].

Chronic low‐grade inflammation increases cancer cells’ ability to invade and metastasize. For example, inflammatory factors such as TNF‐α and IL‐6 promote tumor cell invasion and metastasis by activating the epithelial–mesenchymal transition pathway [118]. Due to these mechanisms, obesity‐related chronic low‐grade inflammation has become a key area of research linking obesity and cancer. Targeting these inflammatory pathways may lead to new cancer prevention and treatment strategies.

#### 3.1.2 Insulin Resistance and Carcinogenesis

Obesity is a key driver of insulin resistance, primarily through excessive adipose tissue expansion, chronic low‐grade inflammation, elevated free fatty acids, and abnormal adipokine secretion [119]. These pathological changes impair the phosphorylation of insulin receptor (IR) substrates and disrupt the PI3K/AKT signaling pathway, thereby weakening the metabolic effects of insulin and ultimately leading to insulin resistance [109]. To compensate for insulin resistance, pancreatic β‐cells secrete more insulin, resulting in hyperinsulinemia. Elevated insulin levels, in turn, inhibit the production of IGF‐binding proteins, increasing the concentration of free IGF‐1 [120, 121]. IGF‐1, a potent mitogen, promotes cell proliferation and survival while inhibiting apoptosis via PI3K/AKT/mammalian target of rapamycin (mTOR) and MAPK pathways, fostering tumor initiation and progression [105, 122, 123]. Insulin resistance and hyperinsulinemia are strongly associated with an increased risk of various cancers. Elevated insulin drives tumor proliferation and metabolic reprogramming through IRs or enhanced IGF‐1 activity, particularly in breast, colorectal, and prostate cancers [8]. Furthermore, sustained activation of the PI3K/AKT/mTOR pathway not only facilitates rapid tumor cell division but also regulates glycolytic enzymes, allowing tumor cells to adapt to high metabolic demands. Additionally, elevated insulin levels promote estrogen biosynthesis, thereby increasing the risk of hormone‐related cancers such as breast and endometrial cancers [124]. Collectively, obesity‐driven insulin resistance and hyperinsulinemia accelerate cancer progression through converging metabolic and signaling abnormalities. Based on these mechanisms, targeting the PI3K/AKT/mTOR pathway or mitigating hyperinsulinemia may offer novel strategies for preventing and treating of obesity‐related cancers.

#### 3.1.3 Dysregulated Adipokines in Tumor Biology

Obesity is characterized not only by excessive fat accumulation but also by significant changes in the endocrine function of adipose tissue. Adipose tissue is an active endocrine organ whose adipokine secretion is markedly altered in obesity. Two hallmark changes include elevated levels of leptin and reduced levels of adiponectin. This dysregulated secretion plays a crucial role in cancer initiation and progression [125, 126].

Leptin, a proinflammatory adipokine, is overproduced in obesity. By binding to its receptor, leptin activates multiple signaling pathways, including the JAK/STAT, PI3K/AKT, and MAPK pathways, which directly promote tumor cell proliferation, survival, and angiogenesis [127, 128, 129]. Studies have shown that leptin expression is closely associated with the development of various cancers, such as breast cancer, CRC, and pancreatic cancer. Moreover, leptin exacerbates the TME by increasing the secretion of proinflammatory cytokines, thereby enhancing tumor cell invasion and metastasis [130].

Conversely, adiponectin, an anti‐inflammatory and antitumor adipokine, is markedly reduced in obesity. By binding to its receptors, AdipoR1 and AdipoR2, adiponectin activates signaling pathways such as adenosine monophosphate‐activated protein kinase (AMPK) and peroxisome proliferator‐activated receptor alpha (PPAR‐α), which inhibit tumor cell proliferation and induce apoptosis [131, 132, 133]. Additionally, adiponectin suppresses the NF‐κB signaling pathway, reducing the production of proinflammatory factors and thereby weakening the role of inflammation in cancer development. It can also induce growth arrest and apoptosis in various cell lines by activating AMPK in a p53‐ and p21‐dependent manner [134]. Reduced adiponectin levels are strongly associated with increased risks of cancers such as breast cancer, liver cancer, and gastric cancer [135].

**FIGURE 2:**
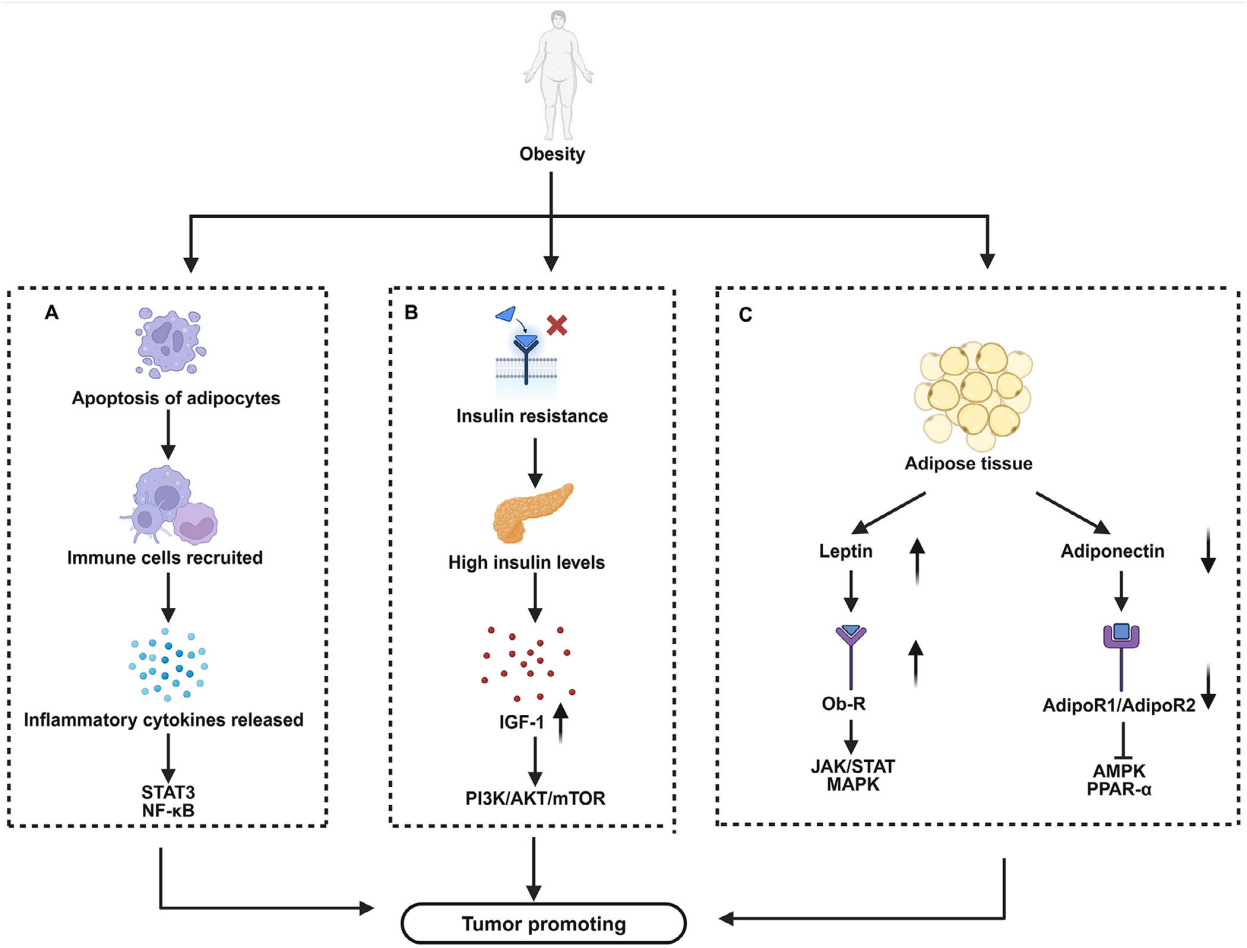
Obesity promotes cancer progression through key mechanisms, including (A) chronic inflammation, (B) insulin resistance, and (C) dysregulated adipokine secretion, which together affect tumor cell proliferation and metastasis. (A) Chronic inflammation: Obesity‐induced adipocyte apoptosis recruits immune cells, leading to the release of proinflammatory cytokines such as TNF‐α and IL‐6. These cytokines activate tumor‐promoting pathways including, STAT3 and NF‐κB, which drive tumor cell proliferation, survival, and invasion. (B) Insulin resistance: Obesity‐associated insulin resistance results in hyperinsulinemia and elevated IGF‐1 levels. IGF‐1 activates the PI3K/AKT/mTOR pathway, promoting tumor growth and enhancing the metabolic adaptability of tumor cells. (C) Dysregulated adipokine secretion: Obesity alters the endocrine profile of adipose tissue, increasing leptin and decreasing adiponectin. Leptin signals through the leptin receptor (Ob‐R) and activates the JAK/STAT and MAPK pathways to promote angiogenesis, invasion, and metastasis. whereas adiponectin signals via AdipoR1/AdipoR2 to activate AMPK and PPAR‐α, supporting anti‐inflammatory and antitumor effects. These obesity‐related mechanisms converge to create a tumor‐promoting microenvironment, facilitating cancer development and progression. *Abbreviations*: TNF‐α, tumor necrosis factor‐alpha; IL‐6, interleukin‐6; STAT3, signal transducer and activator of transcription 3; NF‐κB, nuclear factor‐kappa B; IGF‐1, insulin‐like growth factor‐1; PI3K, phosphoinositide 3‐kinase; AKT, protein kinase B; mTOR, mammalian target of rapamycin; JAK, Janus kinase; MAPK, mitogen‐activated protein kinase; AMPK, adenosine monophosphate‐activated protein kinase; PPAR‐α, peroxisome proliferator‐activated receptor alpha; Ob‐R, leptin receptor; AdipoR1/AdipoR2, adiponectin receptor 1/2.

Overall, altered adipokine secretion reshapes tumor signaling, inflammation, and metabolism (Figure 2). Modulating leptin‐ and adiponectin‐related pathways may thus offer new strategies for preventing and treating obesity‐associated cancers. These insights complement the preceding discussion of insulin resistance and further illustrate how metabolic–endocrine crosstalk drives tumorigenesis in obesity.

#### 3.1.4 The Impact of Obesity‐Induced Fibrosis on Immunotherapy

In obese hosts, the interaction between adipocytes, myeloid cells, and cancer‐associated fibroblasts (CAFs) promotes the extensive deposition and abnormal cross‐linking of collagen, significantly enhancing the desmoplastic reaction and stromal stiffening [136, 137, 138]. This structural and mechanical remodeling forms a physical and topological barrier that restricts T cell migration and hinders the entry of antigen‐presenting cells, as well as effective contact between T cells and tumors due to the disordered arrangement of collagen fibers [139]. Among them, TGFβ‐dependent CAF subtypes further prevent contact between tumor‐infiltrating T cells and cancer cells, driving resistance to ICI [140, 141]. Dense stroma‐induced hypoxia stabilizes HIF‐1α and, in concert with obesity‐related cytokines (such as IL‐6, TNF‐α, and leptin), activates the JAK/STAT3 pathway, upregulates PD‐1/PD‐L1 expression, and suppresses antigen presentation [17, 142]. Obesity leads to more pronounced PD‐1‐dependent T cell exhaustion, which, paradoxically, may render T cells more sensitive to PD‐1 blockade in the context of physical and metabolic suppression. This suggests that the “apparent benefit” is not a pure biological advantage but rather a result of the interplay between dosing/pharmacokinetics and TME remodeling.

### 3.2 Metabolic Dysregulation Induced by Obesity and Its Association With Cancer Progression

#### 3.2.1 Metabolic Abnormalities and Tumorigenesis

Metabolic reprogramming is a hallmark of obesity‐driven carcinogenesis. Among these changes, abnormal glucose metabolism plays a central role. The onset of insulin resistance and sustained hyperglycemia provide tumor cells with a constant energy supply. In a high‐glucose milieu, tumor cells engage in aerobic glycolysis (the Warburg effect), rapidly producing adenosine triphosphate (ATP) and biosynthetic intermediates to sustain proliferation. Although this metabolic reprogramming is less efficient in terms of energy conversion, it provides critical metabolic support for the rapid proliferation of tumor cells. This metabolic adaptation enhances the ability of tumor cells to survive and thrive in adverse environments [143, 144].

The accumulation of glycolytic byproducts is closely linked to this process. These byproducts not only supply the raw materials required for synthesizing membrane lipids and amino acids but also contribute to acidification of the TME. This acidification exacerbates the invasiveness and drug resistance of cancer cells [145].

Beyond glucose, dysregulated lipid metabolism also fuels tumor growth. Obesity‐related dyslipidemia, especially elevated free fatty acids, supplies membrane precursors, signaling molecules, and key energy substrates [146]. Fatty acid oxidation critically sustains tumor proliferation, particularly in breast, pancreatic, and HCC, where fatty acid demand is high [147]. Moreover, lipid metabolism abnormalities are intricately linked to the secretion of proinflammatory cytokines and alterations in immune responses, creating an inflammation‐driven microenvironment that supports tumor growth and metastasis [148].

Evidence indicates that obesity‐induced metabolic filtration stress promotes renal injury and the development of ccRCC. Obesity induces metabolic overload in proximal tubular cells, ultimately leading to chronic renal disease and an increased risk of ccRCC originating from the same proximal tubular cells [149]. Together, these glucose‐ and lipid‐related alterations establish a metabolic landscape that sustains tumorigenesis in multiple organs.

#### 3.2.2 Oxidative Stress and Cancer Initiation

Accumulation of metabolic byproducts, particularly elevated oxidative stress, is another key link between obesity and cancer. Metabolic dysregulation induced by obesity significantly increases the levels of ROS, which in turn cause DNA damage, genetic mutations, and epigenetic alterations. ROS both damage genomic DNA and activate oncogenic pathways such as NF‐κB and PI3K/AKT, promoting tumor proliferation, invasion, and therapy resistance [150].

Interestingly, ROS appear to have a dual role in this process: they not only contribute to tumor initiation but also drive metabolic reprogramming and immune evasion within the TME [151]. Additionally, obesity‐related metabolic byproducts, such as lactate and lipid peroxidation products, suppress the function of immune cells in the TME, potentially altering the properties of the extracellular matrix and thereby facilitating tumor cell invasion and metastasis [152].

**FIGURE 3:**
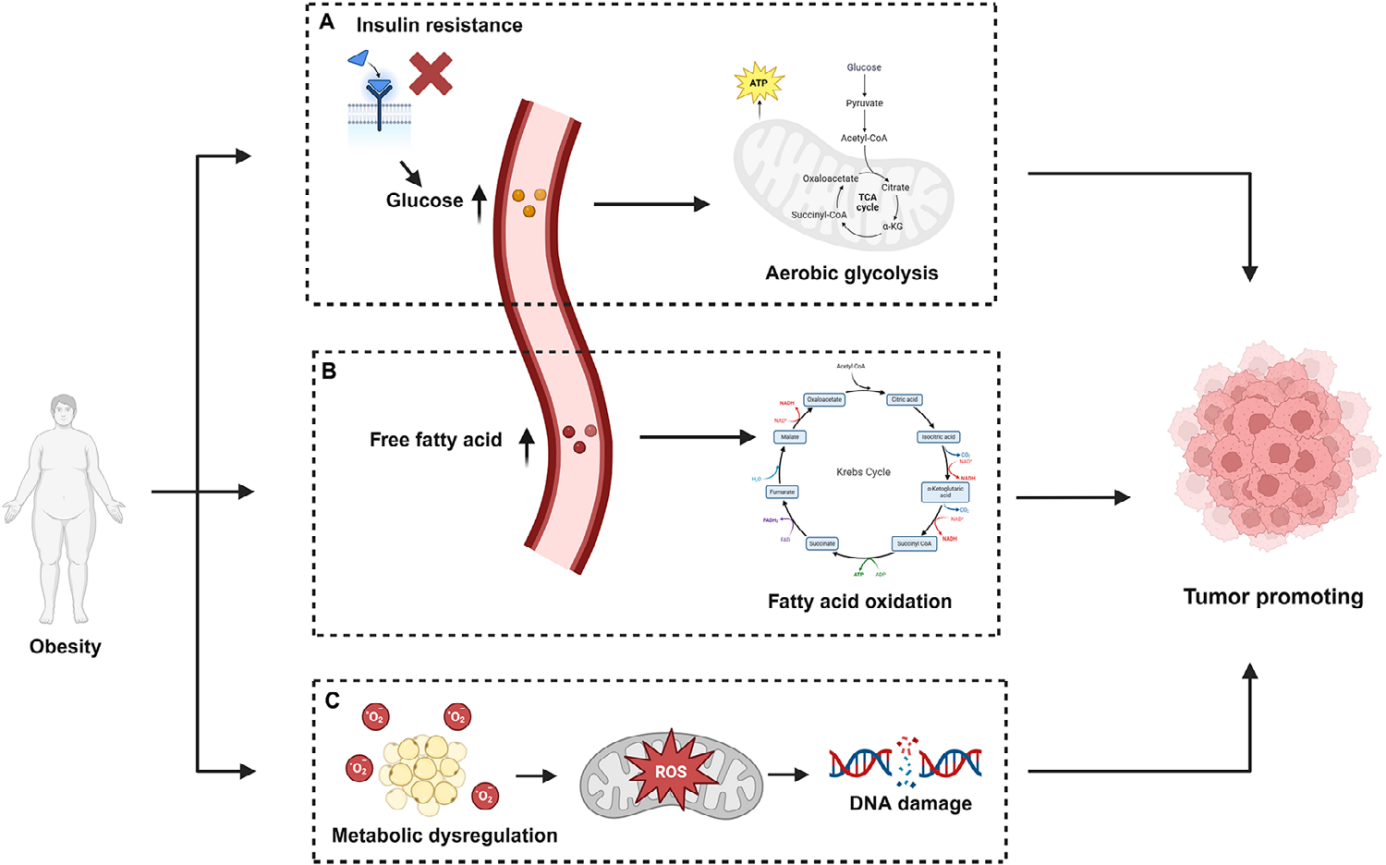
Obesity‐induced metabolic dysregulation promotes cancer progression by creating a tumor‐supportive environment through (A) glucose metabolism, (B) lipid metabolism, and (C) oxidative stress. (A) Glucose metabolism: Insulin resistance in obesity leads to elevated blood glucose levels, which fuel tumor cells through aerobic glycolysis (the Warburg effect). This process rapidly generates adenosine triphosphate (ATP) and biosynthetic intermediates (e.g., amino acids, nucleotides, and lipids), supporting tumor proliferation despite the low energy efficiency of glycolysis. (B) Lipid metabolism: Obesity‐associated dyslipidemia results in elevated free fatty acids, which serve as major energy sources and building blocks for membrane lipids in tumor cells. Fatty acid oxidation sustains tumor proliferation, particularly in breast, pancreatic, and liver cancers. Abnormal lipid metabolism also promotes the secretion of proinflammatory cytokines, establishing an inflammation‐driven microenvironment that enhances tumor growth and metastasis. (C) Oxidative stress: Obesity‐related metabolic dysregulation increases reactive oxygen species (ROS) production, leading to DNA damage, genetic mutations, and epigenetic alterations. ROS further activate tumor‐promoting signaling pathways, such as NF‐κB and PI3K/AKT, driving tumor proliferation, invasion, and resistance to therapy. In addition, metabolic byproducts such as lactate and lipid peroxidation products suppress immune cell function in the tumor microenvironment, remodeling the extracellular matrix and thereby facilitating tumor invasion and metastasis. *Abbreviations*: ATP, adenosine triphosphate; ROS, reactive oxygen species; DNA, deoxyribonucleic acid; NF‐κB, nuclear factor‐kappa B; PI3K, phosphoinositide 3‐kinase; AKT, protein kinase B.

Overall, the metabolic dysregulation caused by obesity provides cancer cells with abundant energy and metabolic intermediates, influencing the initiation and progression of cancer (Figure 3). Targeting these metabolic pathways may offer novel strategies for treating obesity‐associated cancers.

### 3.3 Obesity‐Driven Immunosuppression in the TME

#### 3.3.1 T‐Cell Dysfunction Mediated by Obesity

T‐cell dysfunction is a central mechanism of obesity‐driven immunosuppression. This subsection outlines how obesity reshapes T‐cell immunity to favor tumor progression. Obesity profoundly disrupts immune homeostasis, impairing the function of effector T cells and expanding the number of Treg cells. These changes reduce the body's ability to recognize and eliminate cancer cells [153, 154]. In a high‐fat diet mouse model, CD8⁺ tumor‐infiltrating lymphocytes (TILs) exhibited marked functional impairment, characterized by reduced expression of effector molecules and compromised tumor surveillance. Consequently, tumor cells in obese hosts become harder to recognize and eliminate, increasing cancer risk [17]. Suppressed effector T‐cell activity within the obese TME facilitates immune evasion and tumor progression. Together with an expanded Treg cell population, these changes foster immune tolerance and support tumor growth and metastasis [149]. In addition to the accumulation of immunosuppressive cells, solid tumors can also negatively affect TILs through the expression of inhibitory immune checkpoint molecules by tumor cells and leukocytes, as well as nutrient deprivation and the release of hypoxia‐related factors and immunosuppressive molecules [155]. Collectively, these mechanisms illustrate how obesity undermines antitumor T‐cell responses, setting the stage for the broader immunosuppressive landscape discussed in the following sections.

#### 3.3.2 Accumulation and Activation of TAMs

The immunosuppressive effects of obesity are also strongly manifested through the accumulation and activation of TAM, a key type of immunosuppressive cell in the TME. Chronic low‐grade inflammation characteristic of obesity drives TAM infiltration and expansion, thereby weakening antitumor immunity. These macrophages secrete proinflammatory and protumorigenic mediators, such as IL‐6, TNF‐α, and TGF‐β, which not only promote tumor cell proliferation and metastasis but also inhibit the effector functions of CD8⁺ T cells [156]. This process not only exacerbates immune evasion but also facilitates angiogenesis, metabolic reprogramming, and the establishment of an immunosuppressive microenvironment [157, 158].

In parallel, obesity fosters the recruitment and activation of MDSCs, another key immunosuppressive population. Mechanistically, obesity enhances MDSC maturation and protumor activity by upregulating inducible nitric oxide synthase through the Notch signaling pathway [159]. In a diet‐induced obese breast cancer mouse model, elevated intratumoral C‐X‐C motif ligand 1 (CXCL1) expression further recruited C‐X‐C motif chemokine receptor 2 (CXCR2)⁺ granulocytic MDSCs (G‐MDSCs), whose accumulation triggered CD8⁺ T‐cell apoptosis and contributed to resistance to immunotherapy [160]. Moreover, obesity elevates circulating IL‐6, granulocyte colony‐stimulating factor (G‐CSF), and granulocyte–macrophage colony‐stimulating factor (GM‐CSF), which in turn upregulate immune checkpoint molecules cytotoxic T‐lymphocyte antigen 4 (CTLA‐4) and PD‐L1, thereby further suppressing T‐cell cytotoxicity and amplifying tumor immune escape [161].

Collectively, these obesity‐driven alterations in TAMs and MDSCs create a feed‐forward loop of inflammation and immune suppression, establishing a TME that favors cancer progression and impairs the efficacy of ICIs.

#### 3.3.3 Immune Dysregulation Induced by Obesity Across Cancer Types

Immune dysregulation caused by obesity is evident in multiple cancer types. For example, the development of ccRCC is closely associated with immune system dysregulation triggered by obesity. Studies have demonstrated that in obese patients, the immune microenvironment of renal tumors is characterized by TAM accumulation and effector T‐cell suppression, allowing renal cancer cells to evade immune surveillance and invade surrounding tissues [123, 162]. Similar immune dysregulation has been observed in CRC, where obesity alters the gut immune microenvironment and promotes the accumulation of immunosuppressive cells, increasing the risk of tumor development and metastasis [158, 163]. In malignant melanoma, immune evasion is also closely linked to obesity, which promotes ROS accumulation and expands tumor‐associated immunosuppressive cells, further enhancing immune tolerance and therapy resistance [164].

Recent studies suggest that modulating obesity‐related immune responses may open new therapeutic avenues for obesity‐associated cancers. For example, targeting TAMs or restoring effector T‐cell function has been proposed to overcome immune evasion and improve antitumor immunotherapy outcomes. Additionally, some studies suggest that addressing obesity‐induced metabolic reprogramming, such as the use of AMPK activators or PPAR‐α inhibitors, may alter the immune status of the TME and lead to breakthroughs in cancer treatment [165, 166].

**FIGURE 4:**
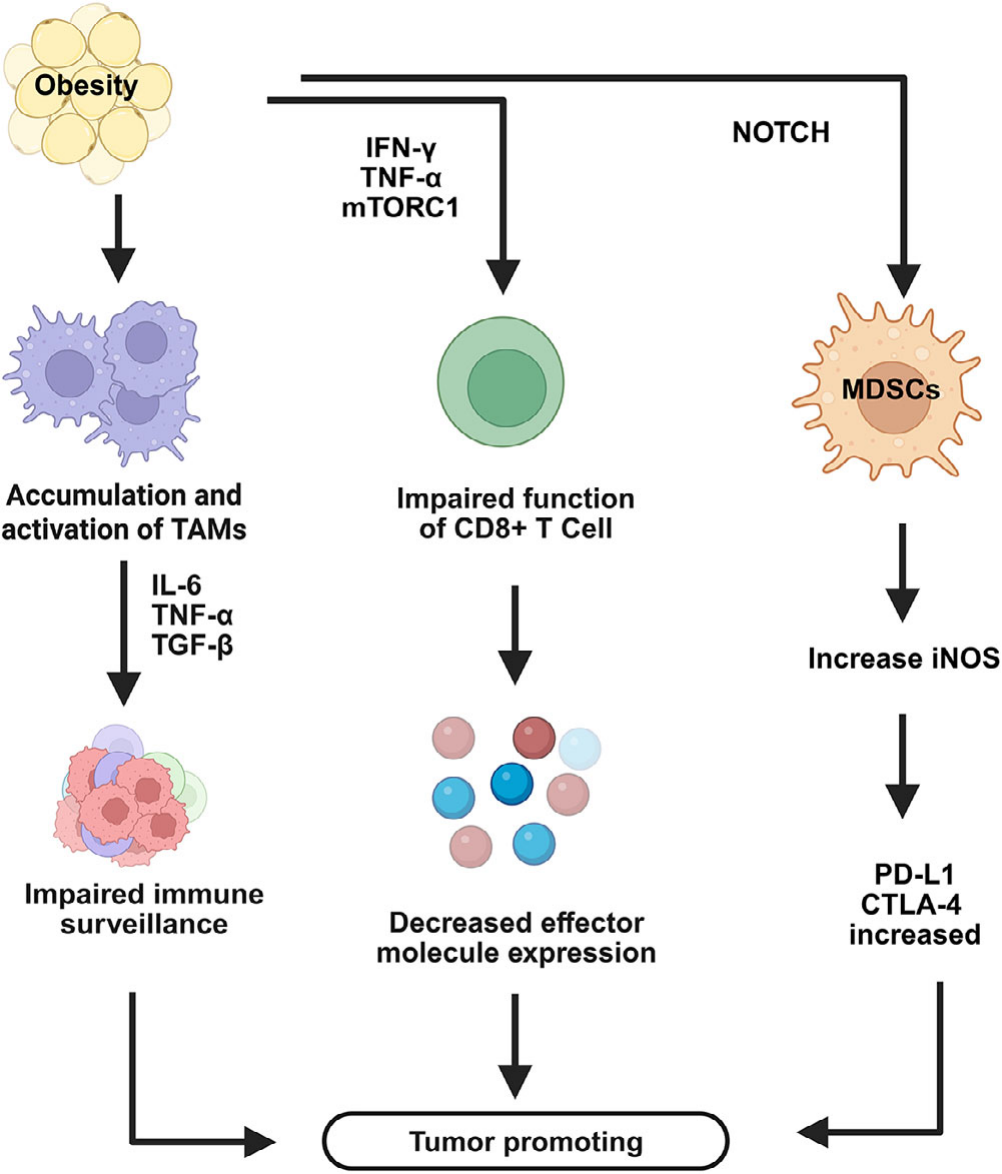
Obesity induces chronic low‐grade inflammation and metabolic alterations that profoundly reshape the tumor immune microenvironment. Excess adiposity drives the accumulation and activation of tumor‐associated macrophages (TAMs), which secrete interleukin‐6 (IL‐6), tumor necrosis factor‐alpha (TNF‐α), and transforming growth factor‐beta (TGF‐β), thereby impairing immune surveillance and fostering tumor growth. Concurrently, myeloid‐derived suppressor cells (MDSCs) are recruited and activated through the Notch signaling pathway, leading to the upregulation of inducible nitric oxide synthase (iNOS) and an enhanced immunosuppressive capacity. Elevated proinflammatory cytokines (e.g., interferon‐gamma [IFN‐γ] and TNF‐α) and mechanistic target of rapamycin complex 1 (mTORC1) activity further impair the cytotoxic function of CD8⁺ T cells, leading to decreased expression of effector molecules. MDSCs and TAMs also promote the upregulation of programmed death‐ligand 1 (PD‐L1) and cytotoxic T‐lymphocyte‐associated protein 4 (CTLA‐4), reinforcing T‐cell exhaustion and immune escape. Collectively, these obesity‐induced changes create a feed‐forward loop of inflammation and immune suppression that promotes tumor initiation, progression, and resistance to immune checkpoint inhibitor therapy. *Abbreviations*: TAMs, tumor‐associated macrophages; MDSCs, myeloid‐derived suppressor cells; iNOS, inducible nitric oxide synthase; PD‐L1, programmed death‐ligand 1; CTLA‐4, cytotoxic T‐lymphocyte‐associated protein 4; IFN‐γ, interferon‐gamma; TNF‐α, tumor necrosis factor‐alpha; mTORC1, mechanistic target of rapamycin complex 1; TGF‐β, transforming growth factor‐beta; CD8⁺ T cells, cluster of differentiation 8 positive T cells.

Obesity‐induced immune dysregulation significantly weakens the body's antitumor immune surveillance ability by impairing the function of immune cells such as T cells and macrophages. This immune dysfunction not only provides fertile ground for cancer immune evasion but also promotes the development and metastasis of various cancers (Figure 4). A deeper understanding of these mechanisms and targeted immune interventions may offer promising new therapeutic strategies for treating obesity‐related cancers.

## 4 Influence of Obesity on Tumor and Immune Dynamics in the Context of ICI Therapy

Immune checkpoints, particularly the PD‐1/PD‐L1 axis, play a pivotal role in tumor immune evasion. PD‐1, an immune checkpoint molecule expressed on T cells, inhibits T‐cell activation when it binds to PD‐L1, which is expressed on tumor cells, allowing tumor cells to evade immune detection. Although obesity‐related chronic low‐grade inflammation usually promotes immunosuppression and tumor escape, several studies show that obese patients often respond better to ICIs. This paradoxical phenomenon may be attributed to obesity‐induced changes in the immune microenvironment, which interact with the mechanisms of ICIs to activate antitumor immune responses. This section examines how metabolic and immune changes in obesity reshape the TME and influence responses to immune checkpoint blockade.

### 4.1 Metabolic Features of Obesity and the Response to Immune Checkpoint Blockade

The metabolic characteristics of tumors, particularly the upregulation of oxidative phosphorylation (OXPHOS) and glycolysis, often create hypoxic microenvironments that drive resistance to ICIs [167, 168]. A study analyzing tissues from metastatic malignant melanoma patients classified by BMI used gene set enrichment analysis and revealed that tumors in overweight and obese patients presented lower OXPHOS and glycolysis levels [19]. These metabolic patterns may increase the sensitivity of tumors to ICIs. In the context of ICI treatment, reduced OXPHOS and glycolysis levels may support antitumor T‐cell functionality by limiting the accumulation of immunosuppressive factors in the TME.

Obesity may also influence the effectiveness of tumor immunotherapy by altering fatty acid metabolism. Fatty acid metabolism supplies key energy substrates that support tumor growth, therapy resistance, and immune modulation. In a study of 126 patients with early‐ to mid‐stage ccRCC, obese patients presented lower expression levels of fatty acid synthase (FASN) but higher expression levels of pathways related to fatty acid metabolism and oxidation [169]. These findings suggest that tumor cells in obese patients may rely on alternative metabolic pathways to meet their energy demands, rather than relying entirely on fatty acid synthesis.

Another study of 61 patients with metastatic ccRCC revealed that FASN gene expression was negatively correlated with BMI, and patients with higher FASN expression levels had shorter OS [170]. These findings suggest that changes in FASN expression reflect metabolic reprogramming in tumor cells in response to obesity. Tumors in obese hosts may therefore reduce de novo fatty acid synthesis and depend on alternative lipid metabolic routes to sustain growth. Collectively, these findings show that obesity reshapes tumor energy metabolism—lowering OXPHOS and glycolysis while redirecting fatty acid utilization—which may increase ICI sensitivity and influence long‐term outcomes.

### 4.2 Tumor Immune Microenvironment Alterations and Their Impact on Immune Checkpoint Blockade

Obesity not only affects tumor cells directly but also alters the TME, particularly the immune infiltration and hypoxic state of peritumoral adipose tissue. A study analyzing transcriptomic differences in their peritumoral adipose tissue between obese and normal‐weight patients revealed that obese patients had increased immune infiltration and hypoxia levels in peritumoral adipose tissue [14]. These findings suggest that peritumoral adipose tissue in obese patients may serve as an immune cell reservoir, potentially contributing to the “obesity paradox” in cancer therapy. The interaction between tumors and surrounding adipose tissue has potential clinical relevance and warrants further exploration.

Leptin has been shown to regulate both innate and adaptive immune responses and may therefore influence the efficacy of immunotherapy. Studies have demonstrated that although transplanted B16 melanomas grow more rapidly in obese mice, tumor growth is more effectively controlled when T cells lack the leptin receptor (Ob‐R), a phenomenon closely associated with reduced PD‐1 expression on CD8⁺ T cells [171]. Studies indicate that obesity induces increased PD‐1 expression on T cells through leptin‐dependent mechanisms, impairing T‐cell function and leading to T‐cell exhaustion. However, this also makes tumor cells more responsive to immune checkpoint inhibition therapy. In a diet‐induced obesity mouse model, anti‐PD‐1 monotherapy significantly increased survival [17]. Further investigations identified STAT3 as the critical mediator linking leptin signaling to PD‐1 expression [172]. Specifically, leptin enhances STAT3 activity, which in turn promotes the upregulation of PD‐1 on T cells [171].

Another study on the relationship between obesity and T cells proposed that obese patients demonstrate better responses to ICIs than nonobese patients do because obesity‐associated immune editing enhances tumor immunogenicity, increasing sensitivity to ICI s and improving therapeutic outcomes [173].

The role of obesity in the TME is also reflected in TAMs. Obesity alters TAM phenotypes through metabolic reprogramming and chronic low‐grade inflammation, promoting their polarization toward the immunosuppressive M2 phenotype. These M2 macrophages secrete anti‐inflammatory factors, such as IL‐10 and TGF‐β, which suppresses effector T‐cell function and facilitates tumor immune evasion and resistance. However, ICIs can target immunosuppressive macrophages or alter the immune cell ratio in the TME to restore immune responses and enhance T‐cell‐mediated tumor clearance. A study revealed that obesity selectively induces PD‐1 expression on TAMs. Obesity‐related type I inflammatory cytokines (e.g., IFN‐γ, TNF, leptin, insulin, and palmitic acid) promote TAM PD‐1 expression through mechanistic target of rapamycin complex 1 (mTORC1) and glycolysis‐dependent mechanisms. This mechanism may drive rapid tumor growth but also enhances the response of obese patients to PD‐1 checkpoint therapy by reversing macrophage‐induced immunosuppression and increasing T‐cell activity, thereby improving the effectiveness of immunotherapy [16]. Moreover, obesity promotes the recruitment of MDSCs, which in turn upregulate the expression of CTLA‐4 and PD‐L1, thereby suppressing T‐cell‐mediated antitumor immunity [161]. Paradoxically, this immunosuppressive state may also enhance the responsiveness of obese patients to PD‐L1‐targeted checkpoint blockade.

Obesity induces systemic chronic low‐grade inflammation, and the neutrophil‐to‐lymphocyte ratio (NLR), calculated from peripheral neutrophil and lymphocyte counts, has been proposed as a novel marker of systemic inflammation that reflects the balance between host inflammatory status and immune responses in cancer patients [174]. NLR has emerged as an important determinant of ICI outcomes. As the most abundant leukocytes in circulation, neutrophils can readily migrate to various tissues and accumulate within diverse tumor types, where they exert distinct and sometimes opposing functions [175, 176]. For example, in murine models responsive to ICI therapy, a significant increase in tumor‐associated neutrophils has been observed [177]. In contrast, clinical studies have reported that elevated NLR is associated with a poor prognosis [178]. Mechanistic investigations suggest that, during tumor progression, mature high‐density neutrophils (HDNs) in the peripheral circulation may convert into protumorigenic low‐density neutrophils (LDNs). LDNs are composed of MDSCs and mature neutrophils derived from HDNs through a TGF‐β‐dependent mechanism [179], which may partly explain the apparent contradiction. Together, these findings underscore the significant heterogeneity between circulating and tumor‐infiltrating neutrophils. Overall, no definitive evidence has yet established that obesity‐driven inflammation is predominantly mediated by neutrophil accumulation. Nevertheless, neutrophils clearly exert multifaceted roles in tumor immunity, which are tightly regulated by a variety of signals within the TME.

**FIGURE 5:**
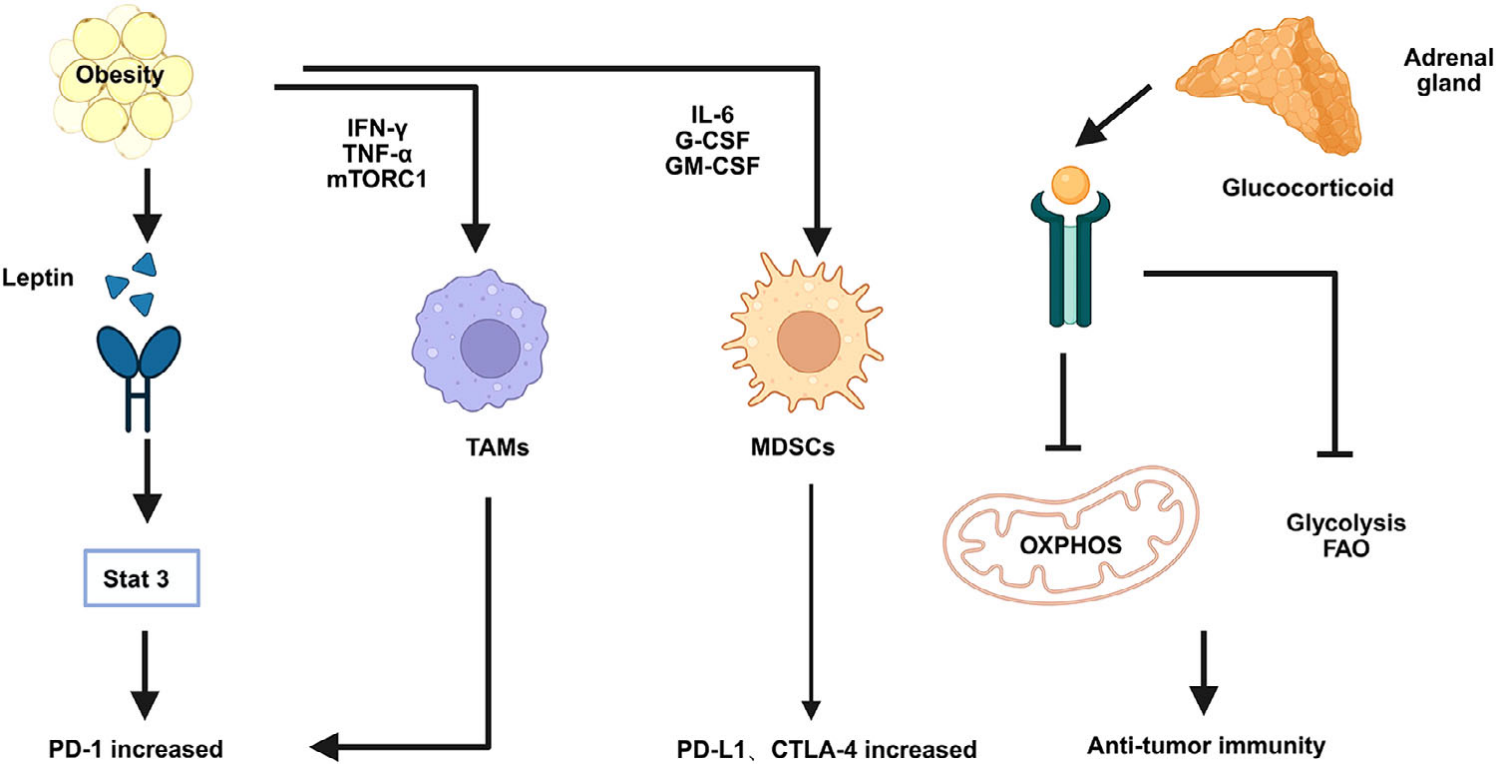
Obesity induces systemic chronic low‐grade inflammation and metabolic reprogramming that remodels the tumor microenvironment (TME) and influences immune checkpoint inhibitor (ICI) responses. Leptin secreted by hypertrophic adipocytes activates the signal transducer and activator of transcription 3 (STAT3) signaling pathway, thereby increasing programmed cell death protein 1 (PD‐1) expression on CD8⁺ T cells and promoting T‐cell exhaustion. Paradoxically, this also enhances tumor sensitivity to PD‐1 blockade. Obesity‐related type I inflammatory cytokines (e.g., interferon‐gamma [IFN‐γ], tumor necrosis factor‐alpha [TNF‐α]) activate mechanistic target of rapamycin complex 1 (mTORC1) in tumor‐associated macrophages (TAMs), driving PD‐1 upregulation and immunosuppressive polarization, while interleukin‐6 (IL‐6), granulocyte colony‐stimulating factor (G‐CSF), and granulocyte–macrophage colony‐stimulating factor (GM‐CSF) recruit myeloid‐derived suppressor cells (MDSCs) that upregulate programmed death‐ligand 1 (PD‐L1) and cytotoxic T‐lymphocyte‐associated protein 4 (CTLA‐4), further suppressing effector T‐cell activity. In parallel, glucocorticoids released from the adrenal gland suppress antitumor immunity by inhibiting mitochondrial oxidative phosphorylation (OXPHOS) and reducing glycolysis and fatty acid oxidation (FAO). Collectively, these obesity‐driven changes create an immune milieu that supports tumor growth yet renders tumors more responsive to PD‐1/PD‐L1 checkpoint inhibition. *Abbreviations*: TAMs, tumor‐associated macrophages; MDSCs, myeloid‐derived suppressor cells; PD‐1, programmed cell death protein 1; PD‐L1, programmed death‐ligand 1; CTLA‐4, cytotoxic T‐lymphocyte‐associated protein 4; IFN‐γ, interferon‐gamma; TNF‐α, tumor necrosis factor‐alpha; STAT3, signal transducer and activator of transcription 3; mTORC1, mechanistic target of rapamycin complex 1; G‐CSF, granulocyte colony‐stimulating factor; GM‐CSF, granulocyte–macrophage colony‐stimulating factor; OXPHOS, oxidative phosphorylation; FAO, fatty acid oxidation; ICI, immune checkpoint inhibitor; TME, tumor microenvironment; IL‐6, interleukin‐6; CD8⁺ T cells, cluster of differentiation 8 positive T cells.

Obesity significantly influences tumor metabolism and the immune microenvironment, both of which interact with ICIs. Obesity‐induced metabolic reprogramming, including reduced OXPHOS and glycolysis levels, as well as alterations in fatty acid metabolism, may enhance ICI sensitivity. Additionally, obesity‐induced changes in immune cells, such as increased PD‐1 expression on T cells and TAMs, create a unique immune microenvironment that paradoxically improves the efficacy of checkpoint inhibition therapy (Figure 5). Further research into these mechanisms may provide novel therapeutic strategies for treating obesity‐associated cancers.

## 5 Controversies and Challenges Surrounding the Obesity Paradox in Cancer

Despite growing evidence of the obesity paradox in various cancer types, its interpretation remains controversial due to multiple biological and methodological complexities. This section discusses the key complexities influencing our understanding of the obesity paradox, including methodological biases, limitations of obesity metrics, and individual differences such as immune‐related toxicity and sex‐specific factors. These challenges underscore the need for more precise, mechanism‐driven research to clarify the role of obesity in cancer immunotherapy.

### 5.1 Complexities in Establishing Causality and the Role of Bias

The causal relationship between obesity and ICI treatment outcomes remains complex. While studies suggest a correlation between obesity and better ICI prognosis, causality is difficult to establish owing to challenges such as unaccounted confounders and collider bias. Many studies lack causal inference tools, such as directed acyclic graphs, to properly control for these factors, risking incorrect attribution of causal links [180, 181].

Confounding variables, such as metabolic diseases, smoking, and disease progression, can influence both BMI and treatment outcomes. For example, in RCC patients, smoking affects BMI, survival, and RCC itself, while BMI‐related metabolic conditions also impact immunotherapy efficacy [170, 182, 183]. Without adjusting for these factors, BMI may appear falsely protective.

Another issue is the reliance on single‐timepoint BMI measurements, which ignore weight changes during treatment, such as cancer‐related cachexia or therapy‐associated weight gain. Weight gain during therapy is linked to improved PFS, highlighting the need to account for dynamic BMI changes [184, 185, 186].

Future studies should incorporate multitimepoint BMI assessments to better capture the impact of dynamic weight changes on ICI outcomes and clarify the causal relationship [187, 188].

At the same time, in early ICI clinical trials, the drug dosage was determined based on the patient's weight (mg/kg), which may lead to a higher total drug exposure in obese individuals and is one of the confounding factors affecting treatment outcomes. Ahmed et al. [189] explored the association between BMI, dosing strategy, and the efficacy of ICI s, including a total of 297 patients, of whom 204 (69%) received fixed‐dose ICI treatment and 93 (31%) received weight‐based ICI dosing treatment. The results showed that the improved prognosis in overweight individuals was limited to those receiving weight‐based ICI dosing treatment (PFS HR 0.53; *p* = 0.04 for weight‐based vs. HR 0.79; *p* = 0.2 for fixed‐dose; OS HR 0.56; *p* = 0.03 for weight‐based; vs. HR 0.89; p = 0.54 for fixed‐dose) [189]. This study seems to suggest that the good prognosis of obese patients is entirely due to their use of more drugs. However, ICI drugs are significantly different from other anticancer drugs, and their mechanism of action is indirect and does not follow a direct dose–efficacy relationship [190, 191]. Meanwhile, the use of fixed doses to replace weight‐based dosing strategies, in addition to the advantage of being easy to manage uniformly, must be based on comparable efficacy [192]. Although high doses caused by large body weight do not directly affect tumor prognosis, the patient's innate immunity and susceptibility remain important factors affecting ICI efficacy [191], and their relationship with obesity still needs further exploration.

### 5.2 Limitations of BMI as an Obesity Indicator: The Influence of Sarcopenia

BMI is a widely used indicator of obesity, but has notable limitations in reflecting metabolic state and fat distribution. It cannot differentiate between subcutaneous fat and visceral fat, the latter being strongly linked to cancer progression and chronic low‐grade inflammation, which may influence ICI efficacy by activating specific immune pathways [193, 194]. At the same time, studies have also shown that VAT is an independent predictor of improved ICI prognosis and may play a key role [195, 196].

Additionally, BMI does not distinguish between adipose tissue mass and skeletal muscle mass, which is critical for ICI outcomes. Sarcopenia (loss of muscle mass) is an independent risk factor for poor cancer prognosis and poor ICI outcomes [185, 197]. Patients with a high BMI may still experience sarcopenia, which compromises immune function, reduces antitumor responses, and lowers ICI efficacy, highlighting the need to assess skeletal muscle mass in conjunction with BMI [196, 198, 199].

Radiological studies have shown that high VAT and SMD are associated with improved OS in male patients with metastatic RCC. Understanding the obesity paradox requires considering both BMI and sarcopenia in ICI treatment [58, 200].

Sarcopenia is also linked to metabolic syndrome and chronic inflammation, which impair ICI responses. Elevated levels of proinflammatory cytokines, such as TNF‐α and IL‐6, are common in sarcopenic patients and may inhibit ICI efficacy. Nutritional and exercise interventions to improve muscle mass should be explored as strategies to improve ICI outcomes [201, 202, 203].

A retrospective study of 219 patients with various cancers (e.g., colorectal, gastric, esophageal, and hepatocellular) who underwent ICIs revealed that obese patients had prolonged OS (*p* = 0.027). Patients with a high visceral fat index (VFI) had significantly longer OS (48.9 months; 95% CI: 43.5–54.2) than did those with a low VFI (32.0 months; 95% CI: 24.1–39.8; *p* = 0.033).

Sole reliance on BMI can be misleading, as patients with similar BMIs may differ significantly in fat distribution and muscle mass, which directly affect ICI outcomes. However, these different approaches are not mutually exclusive Both a higher body weight and a greater muscle mass are associated with better prognosis, suggesting that good nutrition and a strong physical foundation are key to fighting cancer. However, researchers still recommend incorporating additional body composition metrics, such as WHR, skinfold thickness, and bioelectrical impedance analysis, to assess the effects of different fat depots and muscle masses on prognosis. These metrics offer deeper insights into the obesity paradox and its variation across patients [204].

### 5.3 Obesity and its Role in Immune‐Related Adverse Events

Immunotherapy, particularly ICI treatment, achieves significant antitumor efficacy by enhancing immune responses but can also cause immune overactivation, leading to immune‐related adverse events (irAEs). These events pose a significant challenge and are associated with an impaired immune balance. Patients with elevated BMI receiving PD‐1/PD‐L1 inhibitors have a higher incidence of irAEs [205]. For example, high‐BMI patients are more prone to skin‐related irAEs, possibly because obesity‐related chronic low‐grade inflammation promotes tumor progression and immune overactivation during ICI therapy [206].

A retrospective study of 3772 advanced cancer patients—including malignant melanoma (*n* = 832), RCC (*n* = 1000), NSCLC (*n* = 685), microsatellite instability‐high CRC (*n* = 193), HCC (*n* = 214), Hodgkin's lymphoma (*n* = 342), head and neck cancer (*n* = 236), and urothelial carcinoma (*n* = 270)—found that obesity increased the likelihood of irAEs by 71% (OR = 1.71; 95% CI: 1.38–2.11) [207].

Another study of 374 ICI‐treated patients showed that overweight individuals with fewer metabolic comorbidities faced a significantly higher risk of irAEs. These data suggest that obesity increases immune reactivity and amplifies immune responses through metabolic disorders such as hyperglycemia and hyperlipidemia [208].

Together, these findings highlight a complex interplay between obesity, metabolic status, and ICI‐associated irAEs. Clinicians should assess obese patients’ weight and metabolic risk to develop personalized treatment plans. Close monitoring and tailored management are essential to optimize ICI efficacy and minimize adverse events in this high‐risk group.

### 5.4 The Relationship Between Gender and the Obesity Paradox

Sex differences may play an important role in the obesity paradox. Males and females differ substantially in the development and function of their immune systems [209], and these variations influence both the efficacy of ICIs and immune cell metabolism [210].

Clinical evidence indicates that the TME exhibits distinct sex‐specific characteristics. In NSCLC, female tumors exhibit greater immune cell infiltration, characterized by the enrichment of dendritic cells, CD4⁺ T cells, and B cells. In contrast, male tumors are more frequently characterized by T cell‐excluded phenotypes, reduced expression of human leukocyte antigen class I and II molecules, and a higher prevalence of disruptive alterations in antigen‐presentation pathways, all of which impair the efficiency of neoantigen presentation [211].

Sex hormones further modulate these differences. Estrogen exerts immunostimulatory effects, and adipose tissue serves as an important source of estrogen in obese men [212]. Although most female patients in clinical cohorts are postmenopausal, they still benefit from ICI‐based therapies, suggesting that high systemic estrogen levels are not essential for therapeutic efficacy. In men, estrogen may act positively both through direct immune stimulation and indirectly by lowering androgen levels, which are known to suppress T cell function [213].

Conversely, androgen and AR signaling exert profound immunosuppressive effects in males. AR activity diminishes CD8⁺ T cell stemness by downregulating T cell factor 1, promotes T cell exhaustion by expanding PD‐1⁺T cell immunoglobulin and mucin‐domain containing‐3 subsets, and enhances Treg cell‐mediated immunosuppression via stabilization of Foxp3 expression [214]. Collectively, these mechanisms limit antitumor immunity and reduce ICI efficacy in male patients.

In summary, females typically exhibit stronger baseline immune activity and greater T cell infiltration; however, this heightened activation also increases the risk of irAEs. Males, in contrast, are subject to AR‐driven immunosuppression yet may derive benefit from obesity‐associated increases in estrogen. Previous studies often focused on a single sex, and conclusions about whether males or females gain a greater advantage from obesity in immunotherapy remain inconsistent. These observations highlight the necessity of sex‐stratified analyses to tailor and optimize immunotherapy strategies for both women and men.

## 6 Conclusions and Future Prospects

In summary, substantial evidence supports the existence of the “obesity paradox,” where a higher BMI may be associated with improved survival and enhanced efficacy of ICI therapy in certain cancer types (Table 1). This observation challenges the traditional paradigm that obesity uniformly worsens cancer outcomes. It also underscores the need to distinguish clearly between obesity as an etiologic risk factor for cancer initiation and its more nuanced impact on immunotherapy response.

Although clinicians often recommend maintaining or achieving a standard weight for cancer patients, this review suggests that strict dietary control or exercise regimens may not be suitable for cancer treatment. Weight loss may not necessarily improve cancer outcomes and could even have adverse effects on treatment results. While obesity may increase the risk of irAEs during immunotherapy, weight management for long‐term obese patients should be approached cautiously. Excessive weight loss can negatively affect therapeutic outcomes. Instead, lifestyle modifications such as moderate physical activity, healthy diets, and smoking cessation should be encouraged to support overall health.

The future potential of the obesity paradox lies in developing more refined, personalized treatment approaches. Instead of treating obesity as a homogeneous factor, patients could be stratified based on of specific metabolic features, such as insulin resistance, systemic inflammatory markers, or sarcopenia, to better predict which individuals will benefit most from ICIs. Additionally, the role of obesity‐induced inflammation in ICI efficacy offers opportunities to explore new combination therapies. For example, combining anti‐inflammatory treatments targeting obesity‐related inflammation with ICIs could improve outcomes in metabolically unhealthy patients. This dual strategy could mitigate the negative impacts of metabolic syndrome while preserving or enhancing the beneficial immune activation observed in certain obese patients.

Future research should focus on the dynamic interplay among obesity, metabolic reprogramming, and tumor immunity, particularly on how obesity remodels the immune microenvironment. Advances in single‐cell and spatial omics, metabolomics, and microbiome profiling, combined with artificial intelligence and machine learning approaches, will enable the integration of large‐scale data to uncover predictive biomarkers and actionable metabolic pathways. Such efforts will ultimately guide rational patient stratification and the design of tailored immunotherapy regimens that maximize therapeutic benefit while minimizing risk.

## Author Contributions

T. C. and W. Z. wrote this manuscript and created figures and tables. J. S. and W. L. performed the literature search. Z. W. and F. Y. revised the manuscript. F. Y. and L. W. provided guidance throughout the preparation of the manuscript. All authors read and approved the final manuscript.

## Funding

This research was supported by the Noncommunicable Chronic Diseases‐National Science and Technology Major Project (No. 2023ZD0500102), the National Natural Science Foundation of China (Nos. 82472780, 82072825, 82372883, and 81874093, 82573490, 82373253), the Yizhang Talent Program (No. JCYZRC‐B‐006), the Shanghai “Zhong Zhong Zhi Zhong” Research Center Construction Project (2022ZZ01011), and National Key Laboratory Open Project for Basic Medical Science Innovation (JCKFKT‐MS‐006).

## Ethics Statement

The authors have nothing to report.

## Conflicts of Interest

The authors declare no conflicts of interest.

## Data Availability

The authors have nothing to report.
